# Clinical Application of Spatiotemporal Distributed Source Analysis in Presurgical Evaluation of Epilepsy

**DOI:** 10.3389/fnhum.2014.00062

**Published:** 2014-02-10

**Authors:** Naoaki Tanaka, Steven M. Stufflebeam

**Affiliations:** ^1^Athinoula A. Martinos Center for Biomedical Imaging, Massachusetts General Hospital, Charlestown, MA, USA

**Keywords:** magnetoencephalography, epilepsy, distributed source analysis, spike propagation, minimum norm estimate, epilepsy surgery

## Abstract

Magnetoencephalography (MEG), which acquires neuromagnetic fields in the brain, is a useful diagnostic tool in presurgical evaluation of epilepsy. Previous studies have shown that MEG affects the planning intracranial electroencephalography placement and correlates with surgical outcomes by using a single dipole model. Spatiotemporal source analysis using distributed source models is an advanced method for analyzing MEG, and has been recently introduced for analyzing epileptic spikes. It has advantages over the conventional single dipole analysis for obtaining accurate sources and understanding the propagation of epileptic spikes. In this article, we review the source analysis methods, describe the techniques of the distributed source analysis, interpretation of source distribution maps, and discuss the benefits and feasibility of this method in evaluation of epilepsy.

## Introduction

Magnetoencephalography (MEG) is an important, non-invasive diagnostic tool, which acquires neuromagnetic fields generated in the brain with high spatial and temporal resolution. Clinical usefulness of MEG, especially in presurgical evaluation of epilepsy, is well documented in recent reviews (Stufflebeam et al., 2009; Stufflebeam, 2011). Currently, clinical applications of MEG are divided into two categories: (1) spontaneous brain activity analysis, including epileptic spike mapping, most often for determining an irritative zone, (2) mapping of eloquent cortex, such as primary motor cortex and language area for avoiding postsurgical functional deficits (Stufflebeam et al., 2009; Stufflebeam, 2011).

Source localization of MEG spikes is frequently performed in clinical practice for identifying an irritative zone (Otsubo and Snead, 2001; Chuang et al., 2006; Stufflebeam, 2011), providing spatial information of spike activities at the sensor level. Source analysis of MEG typically incorporates anatomical information derived from each individual’s magnetic resonance imaging (MRI), and calculates the sources of neural activities by applying a certain mathematical model to the measured magnetic fields. These cortical and subcortical sources are visualized on the MRI or MRI-based anatomical atlas, and provide current dipole distribution maps.

Calculating intracranial sources from MEG obtained outside the brain, an example of the inverse problem, is mathematically non-unique and ill-posed. Certain assumptions are necessary for providing the proper source modeling. Thus, many procedures of source analysis have been proposed, such as single dipole, multi-dipole, and distributed source models, which are also applied for the source analysis of scalp electroencephalography (EEG) (for review, see Michel et al., 2004; Plummer et al., 2008).

In this article, we review the source analysis methods, describe the techniques of the distributed source analysis, and discuss the feasibility of this method in evaluation of epileptic spikes.

## Single Equivalent Current Dipole Analysis

**Figure 1:**
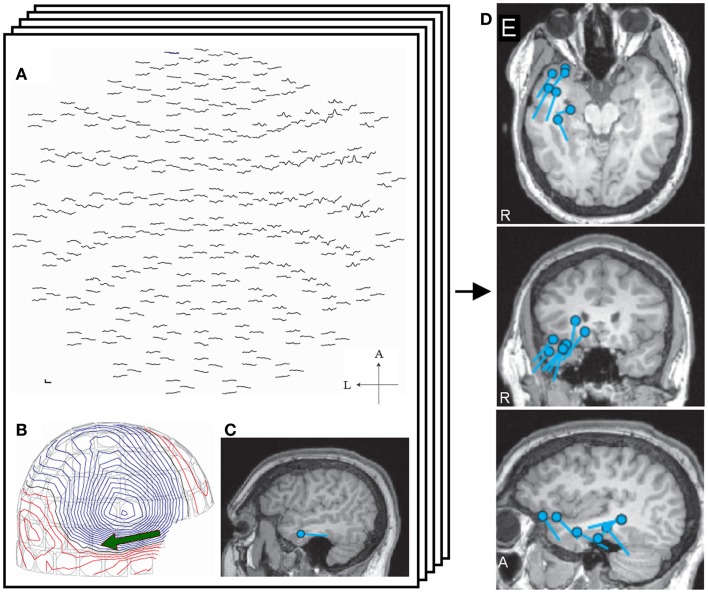
**Single equivalent dipole analysis**. The full view map of the MEG sensor array shows a right temporal spike **(A)**. The contour map of magnetic fields at the peak demonstrates a dipole pattern in the right temporal area **(B)**. The equivalent current dipole (ECD) is projected on the patient’s MRI and is located in the right temporal lobe **(C)**. ECDs obtained from different spikes are collected and mapped on the MRI, providing ECD distribution maps **(D)**.

Single equivalent current dipole (ECD) analysis has been widely used for source localization of epileptic spikes for decades (Otsubo and Snead, 2001; Stefan et al., 2003; Fischer et al., 2005; Chuang et al., 2006). This model assumes that a single dipole source generates all the neuromagnetic fields recorded on the sensors, and is considered physiologically plausible when a limited area of the cortex is synchronously activated. In the analysis, the measured magnetic fields at a given latency are modeled by the best-fitting single dipole. ECDs are typically calculated by using a standard iterative least-square algorithm (Marquardt, 1963; Iwasaki et al., 2002), and several indicators of their reliability are also calculated, such as goodness of fit (GOF) and correlation coefficient. These indicators reflect the concordance between the magnetic fields calculated from the ECD and the actual measurement MEG data. The current dipole moment is represented by the magnitude of the ECD. These indicators and other metrics are used for selecting adequate sources and discarding inadequate ECDs by setting a threshold. Adequate ECDs are mapped on the patient’s MRI, demonstrating the distribution of ECDs (Figure 1). Previous studies have validated ECD analysis in temporal lobe epilepsy (Baumgartner et al., 2000; Iwasaki et al., 2002; Assaf et al., 2004; Reinsberger et al., 2010) and frontal lobe epilepsy (Shiraishi et al., 2001; Genow et al., 2004; Ossenblok et al., 2007). Several studies have shown spatial concordance of ECD distribution and interictal spiking area on intracranial EEG (IEEG) (Mikuni et al., 1997; Oishi et al., 2002).

Although ECD analysis is well established procedure for localizing MEG spikes, a few issues remain. First, the criteria of selecting ECDs may vary from laboratory to laboratory. Single ECDs generally do not provide a GOF of 100%, i.e., 100% of measured magnetic fields can not be explained by a single ECD, due to the oversimplification of this model. Some studies accept ECDs with a GOF >80–90% or correlation coefficient >0.90 (Iwasaki et al., 2002; Genow et al., 2004; Pataraia et al., 2004; Oishi et al., 2006; Knowlton et al., 2009), but other use lower thresholds (Shiraishi et al., 2005a,b, 2011; Reinsberger et al., 2010). The criteria dramatically affect the number of adequate ECDs (Tanaka et al., 2009a), although these thresholds are determined subjectively. Several researchers selected certain sensors for calculating ECDs to obtain adequate ECDs (Iwasaki et al., 2002; Pataraia et al., 2004). Such a process adds another assumption that spikes only appear in a group of restricted sensors. Second, the ECD analysis is sometimes inaccurate (Kobayashi et al., 2005) such as when the signal-to-noise ratio (SNR) is low and the spike is widespread (Shiraishi et al., 2005a; Hara et al., 2007). This may obscure the precise localization of early ictal discharges, which usually have a low SNR (Tanaka et al., 2009a). Third, the single ECD model does not always provide an accurate representation of the time course of the epileptiform discharge. When ECDs are sequentially calculated from the onset to the peak of spike, the SNR is low in the early latency. ECDs obtained from the spike onset may have low GOF values and thus an unstable localization (Kanamori et al., 2013). Several clinical studies calculate ECDs at the spike peak (Iwasaki et al., 2002; Oishi et al., 2006; Reinsberger et al., 2010; Jin et al., 2013) for obtaining a high SNR. However, spikes may be widespread or may propagate, and those ECDs may not accurately identify the onset of the discharge. Moreover, recent studies have proposed that epilepsy is a network disease caused by abnormal neural networks (for review, see Spencer, 2002). Spike propagation is required for understanding the abnormal networks (Tanaka et al., 2010), and advanced analysis procedure is necessary for understanding the spike propagation.

## Spatiotemporal Distributed Source Analysis

Distributed source models assume that a certain amount of cortical patches are simultaneously activated (Dale and Sereno, 1993; Hämäläinen and Ilmoniemi, 1994). This model calculates the source distribution by deploying numerous unit dipoles on the cortical surface. The source space is created by using a realistic head model, such as the boundary elemental model (Hämäläinen and Sarvas, 1989; Oostendorp and van Oosterom, 1989; Crouzeix et al., 1999; Fuchs et al., 2001), obtained by cortical surface reconstruction derived from MRI (Dale et al., 1999; Fischl et al., 1999). Theoretically, the source solution gives concordance between the simulated and measured magnetic fields by adjusting the strength and orientation of unit dipoles (Dale and Sereno, 1993; Hämäläinen and Ilmoniemi, 1994). The source distribution can be calculated at any time points of spike, and from which dynamic activation maps along with the time course can be created. Thus, the source distribution maps represent the cortical activation generated by the spikes both spatially and temporally. The distributed source analysis does not require an assumption on the number of dipoles and thresholds of likelihood parameters as used in the single dipole analysis. However, an infinite number of source distributions can generate a similar magnetic field pattern, and further assumption, such as source distribution with minimum overall intensity (L2-norm), is necessary for determining the optimal solution. Thus, many types of analysis have been proposed based on different assumption and modeling, referred as minimum norm estimate (MNE) (Hämäläinen and Ilmoniemi, 1994), dynamic statistical parametric mapping (Dale et al., 2000), Laplacian weighted minimum norm (LORETA) (Pasqual-Marqui et al., 1994), local autoregressive average (LAURA), and EPIFOCUS (Grave de Peralta et al., 2001). Here, we use the MNE as an example of a distributed source solution.

## MNE Analysis in Practice

This section explains spike analysis with MNE software (www.mne.org), which mainly provides an MNE and dSPM solution algorithm.

### MEG preprocessing – spike selection and clean up

The purpose of preprocessing is to obtain spike data from raw MEG with minimum artifacts. Both individual and averaged spikes can be analyzed by distributed source models. Note that each of these spikes provides identical series of source distribution maps. Visual inspection of spikes is widely performed while automated spike detection is also introduced (Ossadtchi et al., 2004). However, the MEG spike morphology has not been sufficiently described while EEG spikes were well documented in the literature (International Federation of Societies for Clinical Neurophysiology, 1974). A clinical guideline has recommended identifying the MEG spikes based on the principals established for EEG (Bagic et al., 2011). Clarification of MEG spikes will be useful for clinical application of MEG especially in patients with negative EEG findings. Spike selection is critical for obtaining appropriate averaged spikes and spike morphology must be considered.

**Figure 2:**
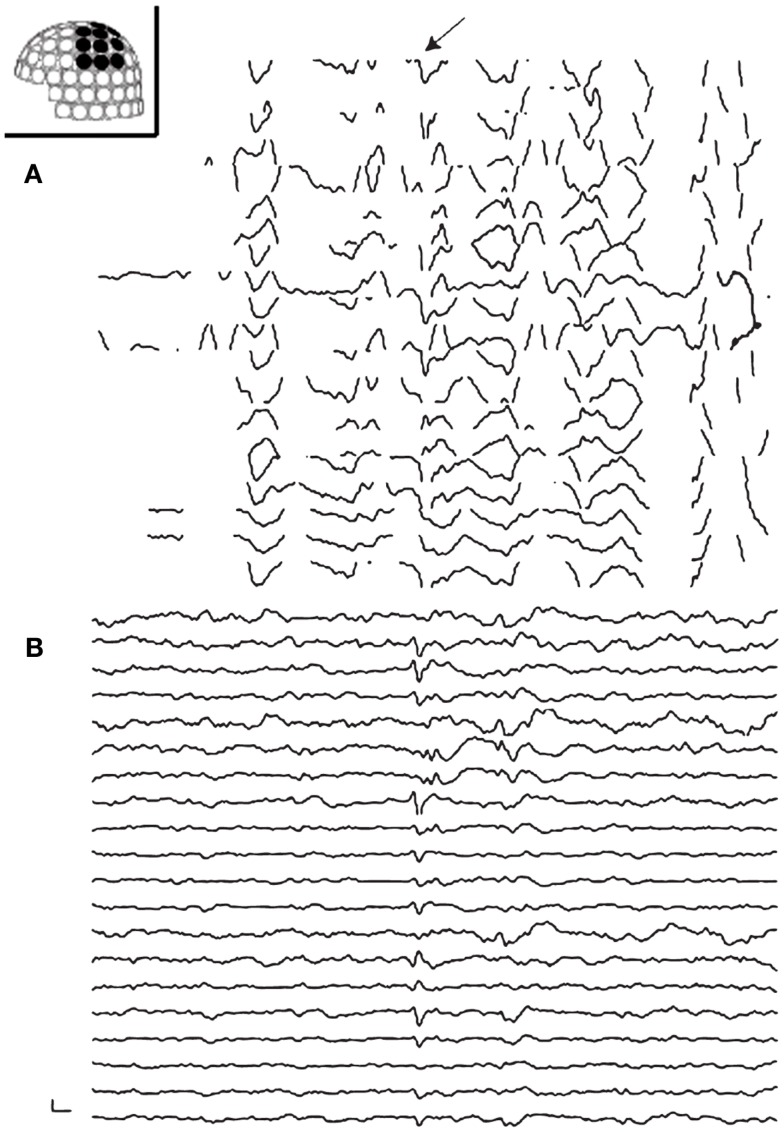
**Preprocessing of MEG with tSSS**. Panels show MEG waveforms in the left centroparietal sensors recorded on a patient with vagus nerve stimulator (VNS). VNS artifacts contaminate the MEG data **(A)**. MEG processed with tSSS shows a prominent spike-and-wave complex **(B)**.

Artifact reduction is also an important issue for calculating adequate sources of spikes. Exclusion of sensors (channels), which contain continuous artifacts, is helpful for avoiding inadequate affection on the source analysis. Independent component analysis may be useful for removing artifacts specific patterns (e.g., ECG) as used on EEG (Kobayashi et al., 2001). Taulu et al. (2004) (Taulu and Simola, 2006; Taulu and Hari, 2009) proposed signal source separation (SSS) and its temporal extension (tSSS). SSS decomposes MEG signals into two components, which are generated from sources inside and outside of the sensor space, and remove the latter component. The temporally extended SSS also considers temporal signal correlation and is widely used in clinical practice (Medvedovsky et al., 2009; Song et al., 2009; Tanaka et al., 2009b; Jin et al., 2013; Kakisaka et al., 2013) (Figure 2). Motion artifacts distort geographic information of the head and sensors, resulting in unreliable source localization. These movements can be compensated by tracking the head position during acquisition, and this technique is useful for analyzing ictal MEG data, where head deviation sometimes occur during seizures (Medvedovsky et al., 2007; Kakisaka et al., 2012).

### MRI preprocessing – creating a head model

Minimum norm estimate constrains source activities to the cortical surface images. The cortical surface is reconstructed from anatomical T1 MRI data, and the reconstruction is the first step of MRI processing. Various software packages, such as Freesurfer (Dale et al., 1999; Fischl et al., 1999) and Brainstorm (Tadel et al., 2011), provide cortical surface reconstructions. The source space is created by using the cortical surface, deploying grid spacing with numerous cortical patches (Crouzeix et al., 1999; Fuchs et al., 2001). Unit current dipoles are distributed in the source space, and the boundary elemental method (BEM) creates a head model for calculating the activation of these dipoles (Hämäläinen and Sarvas, 1989; Oostendorp and van Oosterom, 1989; Crouzeix et al., 1999; Fuchs et al., 2001). A single-layer BEM model is generally used, since the neuromagnetic signals are not affected by the tissue conductivity (Hämäläinen and Sarvas, 1989).

**Figure 3:**
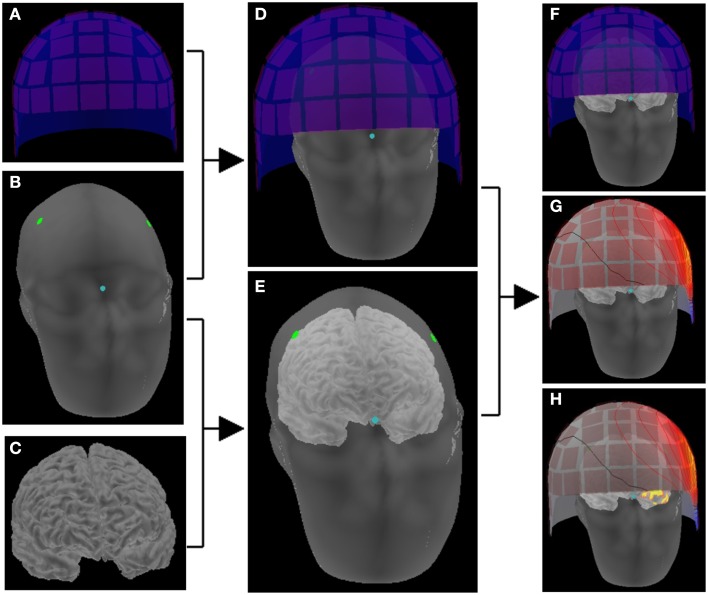
**Schematic representation of making a head model**. The location correlation of sensors **(A)** and head **(B)** is determined by digitization information (dots) and head position indicator in the sensor array **(D)**. The location information of head **(B)** and brain **(C)** is obtained on the reconstructed images of MRI **(E)**. Thus, the brain and the sensor array are co-registered **(F)**, and magnetic fields on the sensor space provides activation maps on the source space **(G)** that consists of the cortical surface **(H)**.

Coregistration of the patient’s MRI with the MEG sensor space is typically performed through the digitization information of the head and head position indicator that generates artificial electric currents in the sensor array. This process estimates the relationship between the source and the sensor spaces (Figure 3).

### Inverse solution, creating source maps

**Figure 4:**
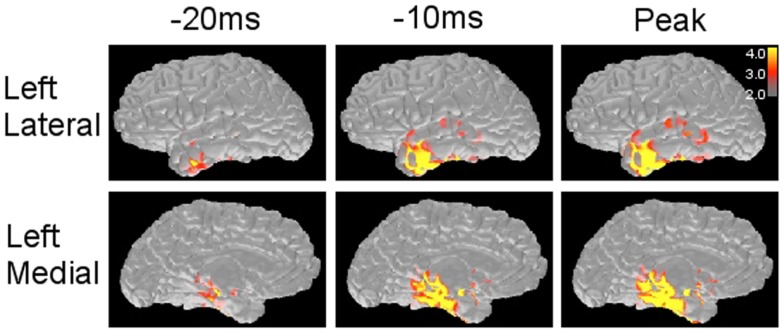
**Minimum norm estimate maps obtained from a left temporal spike**. MNE solution provides source distribution maps along with the time course of spike at different time points. The scale (right corner) demonstrates that the values above 3.0 × 1e−9 and 4.0 × 1e−9 (nA m) are shown as red and yellow, respectively.

The forward solution, which models the magnetic signals generated by unit current dipoles, is obtained by using the coregistration (Hämäläinen and Sarvas, 1989; Oostendorp and van Oosterom, 1989). Inverse solution is calculated based on the forward solution, mapping the strength of each unit dipole (Dale and Sereno, 1993; Dale et al., 2000). Source activation is projected on the cortical surface by applying a certain threshold, showing the source strength with different colors (Figure 4). The strength and extent of activation change along with the time course (Figure 4).

## Interpretation of Distributed Source Maps

**Figure 5:**
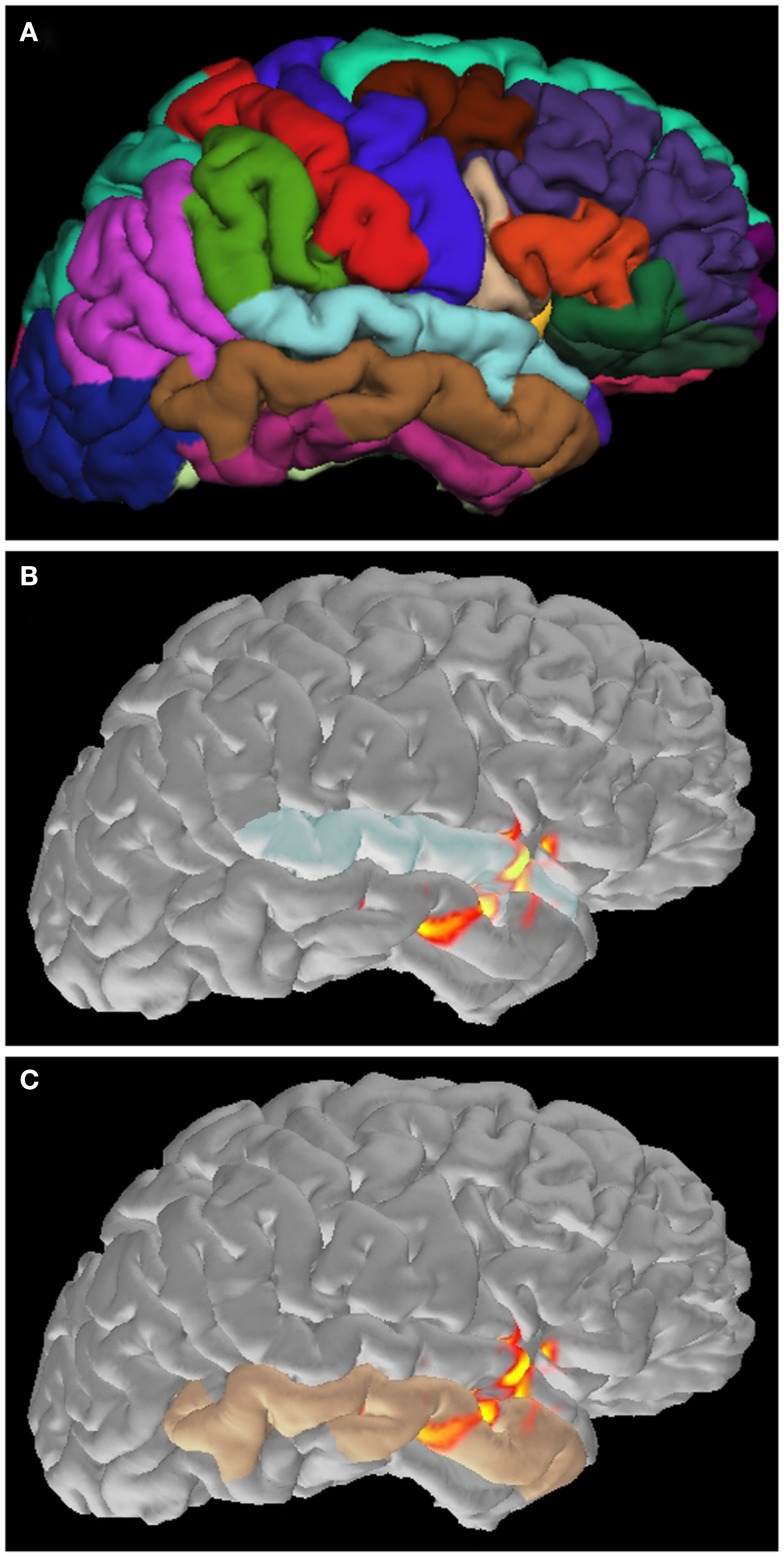
**Application of cortical parcellation**. Cortical parcellation is obtained by Freesurfer **(A)**. The parcellation is useful to understand that the spike activation is mainly located in the superior **(B)** and inferior **(C)** temporal gyrus.

Distributed source maps can be examined both spatially and temporally; these maps estimate the spatial source distribution and its time course. A cortical parcellation, provided by Freesurfer (Dale et al., 1999; Fischl et al., 1999), is helpful for understanding detailed anatomical location of activation (Figure 5). More importantly, spatial source distribution is considered representing to the cortical extent of spike involvement; activation in a small cortical area suggests that the spiking area is restricted (Hara et al., 2007; Tanaka et al., 2013b). Widespread cortical activation, extending over two lobes or bilaterally, may reflect a large abnormal neural network associated with epilepsy (Shiraishi et al., 2005a,b, 2011).

**Figure 6:**
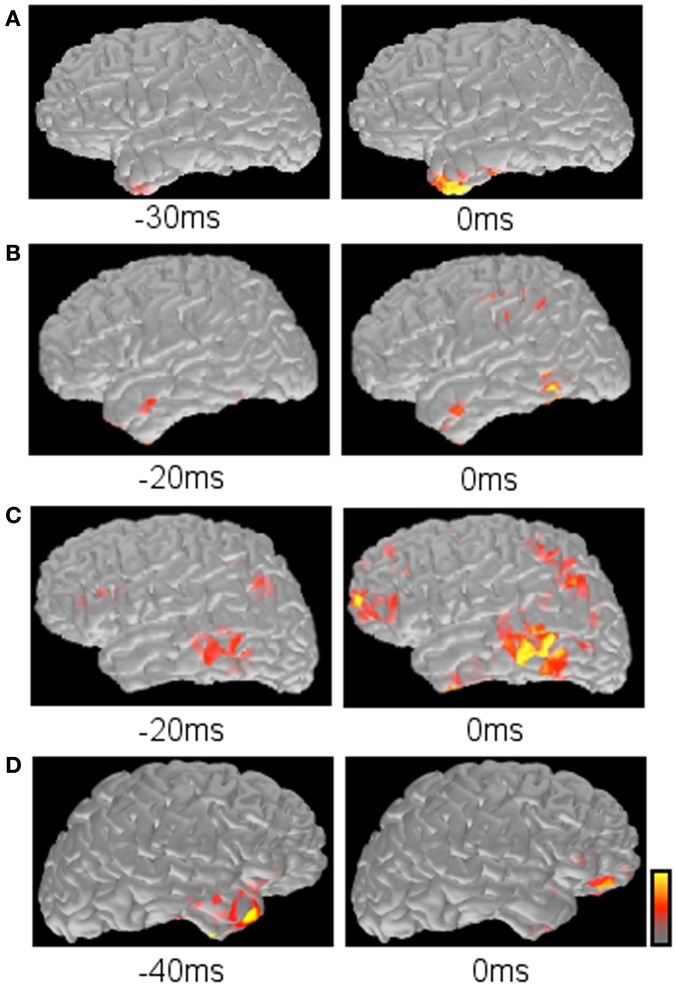
**Typical propagation patterns suggested by MNE maps**. **(A)** Restricted onset and restricted peak activation in the left anterior temporal lobe. **(B)** Restricted onset in the left anterior temporal lobe and widespread peak in the left posterior temporal and parietal lobes. **(C)** Widespread onset and widespread peak in the left frontal, temporal, and parietal lobes. **(D)** Restricted early activation in the right anterior temporal lobe and late activation in the right inferior frontal lobe.

Time course of source activation provides a way to evaluate spatial source distribution at each time point of the spike (Tanaka et al., 2010, 2012). The interpreter can trace the activation pattern from the spike onset to the peak. The changing pattern along with the time course may reflect physiologic spiking process, such as growing or propagation. Here, several patterns of spatiotemporal source distribution can be recognized; (1) restricted onset activation + restricted peak activation in the onset area, suggesting a highly limited spike involvement, (2) restricted onset activation + widespread peak activation, suggesting broad spike propagation, (3) widespread onset activation + widespread peak activation, suggesting non-localizing, widespread spiking in the cortex, (4) restricted early activation + restricted late activation in the distant area, suggesting an abnormal propagation pathway (Figure 6).

There are still several issues for assuming that the MNE maps accurately show the spike distribution in the whole brain; (1) MEG has different sensitivity to intracranial electric currents depending on regions (de Jongh et al., 2005; Goldenholz et al., 2009), and the information in the region with low sensitivity may be missing. (2) MNE solution does not consider the anatomical connection through white matter tracts. Tractography using diffusion tensor imaging will be useful in combination with MNE maps (Tanaka et al., 2012). (3) There is no established way to determine the threshold objectively, although such a threshold greatly affects the appearance of source maps. Several studies have introduced thresholds which are determined by a quantitative procedure (Gallagher et al., 2012; Tanaka et al., 2013a,b). Further investigation will be necessary to understand how the threshold should be determined.

## Benefits of Distributed Source Maps

Previous studies using distributed source analysis mainly focused on providing the evidence of accurate source localization in comparison with IEEG (Waberski et al., 2000; Zumsteg et al., 2006; Tanaka et al., 2010; Kanamori et al., 2013). There are only a limited number of studies describing the benefit of this method in clinical practice (Tanaka et al., 2009a, 2013b). However, the results of these studies suggest benefits to use this technique in clinical practice.

One of the possible benefits is to obtain accurate spike localization. Distributed source analysis likely provides more reasonable solution than a single dipole model, although there is a localization error still observed in various distributed models (Waberski et al., 2000; Silva et al., 2004; Soufflet and Boeijinga, 2005). Previous studies have shown that MNE (and its derivative, dSPM) provides more accurate source localization than single dipoles, by comparing with single photon emission tomography (Shiraishi et al., 2005a), surgical outcome (Tanaka et al., 2009a), and IEEG (Kanamori et al., 2013) in a small group of patients. By using the distributed source analysis, MEG may contribute to the presurgical evaluation of epilepsy more effectively.

The other benefit is to understand the spike propagation. Some epileptic spikes originate from a restricted onset and propagate to other cortical areas (Alarcon et al., 1994, 1997). In these spikes, source localization of the early phase may be more informative than analyzing the peak for identifying the spike origin. Single dipole method sometimes does not provide adequate sources at the early latency as described above (Kanamori et al., 2013), whereas distributed source analysis provides reliable source distribution which can reconstruct the original, small signals of spikes at the sensor level. This ability is also useful for analyzing ictal MEG, which shows only small discharges in the early phase of seizures (Tanaka et al., 2009a). Distributed source analysis has nicely shown the possible onset of spikes with widespread cortical involvement at the peak in the previous studies (Shiraishi et al., 2005a,b; Kanamori et al., 2013).

## Future Directions

Single dipole method has been potentially investigated and its benefits and limitations are well understood, and now several laboratories are doing similar studies with MNE and other distributed source solutions. Recent studies have validated MNE source distribution with IEEG. Tanaka et al. (2010) demonstrated that MNE analysis of frontotemporal MEG spikes accurately represents spike propagation as observed in IEEG. Frontoparietal and temporoparietal propagation patterns are also consistent between MEG and IEEG in a series of cases (Kanamori et al., 2013). Such validation will be highly desirable in other propagation involving various regions and in patients with various types of epilepsy.

An important distinction between ECD and MNE is that distributed source maps generally show the source localization of one single spike whereas single dipole maps project many dipoles obtained from different spikes. Thus, the single dipole method provides a viewpoint of a spike population, such as “clustered” or “scattered.” Distributed source maps do not have such mapping procedures that are widely used. Therefore, combined use of single dipole maps and distributed source maps is necessary in the current settings of spike analysis. Development of new mapping techniques, which overview numerous distributed source maps, will be useful for analyzing many spike populations.

Another area under active investigation is the surgical implications of distributed source maps. Several studies have demonstrated that MEG affects the planning IEEG placement and interpretation (Sutherling et al., 2008; Knowlton et al., 2009), and correlates with surgical outcomes (Iwasaki et al., 2002) by using a single dipole model. On the other hand, a recent study has shown that these propagation patterns are highly correlated with surgical outcomes in patients with temporal lobe epilepsy by using MNE (Tanaka et al., 2013b). Comparison of these techniques regarding with surgical outcomes is now becoming better understood.

## Conclusion

Spatiotemporal distributed source analysis is highly useful for understanding epileptic spikes. It provides more accurate source localization than a single dipole model in some situations, and may be informative in presurgical evaluation of epilepsy. However, further observations are necessary for establishing its usefulness in clinical practice.

## Conflict of Interest Statement

The authors declare that the research was conducted in the absence of any commercial or financial relationships that could be construed as a potential conflict of interest.
